# Health Insecurity and Occupational Risk Factors Among Urban Waste Collectors in Dhaka City, Bangladesh: A Cross‐Sectional Study

**DOI:** 10.1002/hsr2.72281

**Published:** 2026-04-05

**Authors:** Kamrunnahar Koli, Salma Begum, MD. Abujar Gifary Efat

**Affiliations:** ^1^ Department of Sociology Dhaka International University Dhaka Bangladesh; ^2^ Department of Sociology University of Dhaka Dhaka Bangladesh

**Keywords:** Bangladesh, health insecurity, occupational health, personal protective equipment, urban sanitation workers, waste collectors

## Abstract

**Background and Aims:**

Urban waste collectors play an essential role in maintaining urban sanitation, yet they remain one of the most occupationally vulnerable groups in rapidly urbanizing cities in low‐ and middle‐income countries. In Bangladesh, evidence on their occupational health insecurity and access to healthcare services remains limited. This study aimed to assess health insecurity among urban waste collectors in Dhaka City by examining occupational exposures, safety practices, health outcomes, and access to healthcare services.

**Methods:**

A cross‐sectional quantitative study was conducted in 2025 among 40 urban waste collectors working under Dhaka North and Dhaka South City Corporations. Data were collected through face‐to‐face semi‐structured interviews with waste collectors. Descriptive statistics, *χ* ^2^ tests, Pearson's correlation, and exploratory multiple linear regression were used to examine associations between occupational factors and the Health Insecurity Index.

**Results:**

Work‐related health problems were highly prevalent among the respondents. Respiratory disease was reported by 35/40 respondents (87.5%), skin infections were reported by 30/40 respondents (75.0%), allergic conditions by 25/40 (62.5%), and work‐related injuries by 10/40 (25.0%). Longer working hours were associated with higher health insecurity (*r* = 0.58, *p* < 0.001). Only 12/40 workers (30.0%) reported using personal protective equipment (PPE), which was associated with lower health insecurity (*β* = −0.38, *p* = 0.001). Occupational safety training was reported by 9/40 workers (22.5%) and was also associated with lower health insecurity (*β* = −0.29, *p* = 0.03). Only 5/40 respondents (12.5%) reported access to institutional healthcare services, and none reported financial support.

**Conclusion:**

Urban waste collectors in Dhaka City appear to experience considerable health insecurity that may be associated with modifiable occupational and institutional factors. Strengthening occupational health and safety systems, ensuring access to PPE and training, regulating working hours, and expanding healthcare access are important for protecting this essential workforce and supporting sustainable urban waste management.

## 1 Introduction

Rapid urbanization has intensified challenges related to solid waste management and occupational health in low‐ and middle‐income countries (LMICs) [1]. In developing rapidly growing megacities, manual waste collection remains an essential component of urban sanitation systems; however, the workers performing this service often face hazardous working conditions [2]. Dhaka is the capital of Bangladesh and one of the fastest‐growing South Asian megacities, which produces more than 7000 tons of solid waste daily, placing substantial pressure on municipal waste management systems, the labor force responsible for waste collection, and the communities they serve [3]. Municipal and informal waste collectors play a vital role in ensuring urban hygiene and public health, but they are among the most occupationally vulnerable groups because these services are often poorly protected institutionally, have limited technological support, and show weak compliance with occupational health and safety (OHS) regulations [4].

Waste collectors in Dhaka mostly come from socioeconomically marginalized communities and are often employed through precarious work relationships [5]. Their routine work involves direct contact with waste from unsegregated household, industrial, and medical sources, exposing them to multiple physical, chemical, and biological hazards. Empirical studies carried out in Bangladesh have consistently reported a high prevalence of musculoskeletal disorders, respiratory illnesses, dermatological conditions, gastrointestinal diseases, and occupational injuries among waste collectors [6, 7]. These occupational health risks are further complicated by limited access to clean water, sanitation facilities, healthcare services, and employer‐supported safety measures [8]. The vulnerability of waste collectors was further exacerbated in the post‐COVID‐19 period, when waste management systems faced additional disposal challenges with aggravating infectious materials from healthcare facilities and households, coupled with limited access to personal protective equipment (PPE) and formal training [6, 9]. Gender‐based inequalities further compound these risks. Female waste collectors often experience additional economic marginalization and social stigma, and restricted access to protective resources [10].

International evidence suggests occupational health insecurity among waste collectors is a widespread concern in LMICs. Studies conducted in South Asia, Africa, and the Middle East consistently report high levels of work‐related illnesses, injuries, psychosocial stress, and poor hygienic practices among waste workers [11, 12, 13, 14]. Musculoskeletal disorders are among the most commonly reported conditions, often associated with repetitive movements and prolonged manual handling of waste, and poor ergonomic conditions [6]. Respiratory illnesses related to exposure to dust, toxic fumes, and bioaerosols have also been frequently reported, as well as dermatological conditions related to unprotected contact with mixed waste streams [11, 13]. Injuries from sharp objects like broken glass and discarded needles are another indication of poor waste segregation practices and poor occupational safety measures [12]. Across settings, these adverse outcomes are very much linked to poor PPE usage, lack of occupational safety training, and limited access to regular health screening or institutional healthcare support [13, 14].

Beyond the potential for risks in the workplace, proper management of waste has much broader implications for overall public health and the environment. Improper waste disposal is a contributing factor to the spread of infectious and vector‐borne diseases by creating breeding grounds for disease vectors and increasing the risk of dengue and other communicable diseases in urban environments [15]. Poorly handled healthcare waste also poses other threats, including exposure to infectious agents, residues of pharmaceuticals and antimicrobial‐resistant pathogens, which may lead to diseases such as cholera, hepatitis B, respiratory infections and bacteraemia [16]. Environmental contamination through plastic pollution, electronic wastes, hospital effluents, and open dumping practices contributes to long‐term degradation of the air, soil, and water systems that further increase climate‐related vulnerabilities and negate urban environmental sustainability [16, 17].

In spite of the increasing recognition of these challenges, policy and institutional responses often remain fragmented. The literature emphasizes the need for integrated waste management and occupational health policies with mandatory provision of PPE, enforcement of occupational safety training, and regulation of working hours along with regular health monitoring of waste workers [6, 13]. Gender‐sensitive intervention and empowerment programs are also identified as a key area of focus to reduce the disproportionate risks faced by the female waste collectors [10].

Technological innovations such as autoclaving, gasification, pyrolysis and chemical disinfection have been suggested as complementary options to curb pollution in the environment and safe handling of hazardous wastes [15, 16]. However, empirical evidence on this issue in Bangladesh, particularly in Dhaka City, remains limited, especially regarding the combined impact of occupational exposures, institutional support, behavioral practices and access to healthcare that may influence health insecurity of waste collectors. This lack of context‐specific data restricts the development of effective and targeted OHS interventions.

Against this background, the present study aims to assess the level of health insecurity among urban waste collectors in Dhaka City by examining occupational exposures, safety practices, and access to healthcare services. The study further seeks to identify major work‐related health problems and analyze key socio‐environmental, institutional, and behavioral factors associated with health insecurity in this occupational group.

## 2 Methodology

### 2.1 Study Design

A cross‐sectional quantitative study was conducted in 2025 among urban waste collectors working under Dhaka North City Corporation (DNCC) and Dhaka South City Corporation (DSCC) to assess health insecurity and its occupational determinants. This design was appropriate for estimating the prevalence of work‐related health problems and examining associations between occupational exposures, safety practices, institutional factors, and health insecurity at a single point in time. Given the limited empirical evidence on this population in Bangladesh and the modest sample size, the study was designed as an exploratory investigation rather than a causal analysis.

### 2.2 Study Area

**Figure 1:**
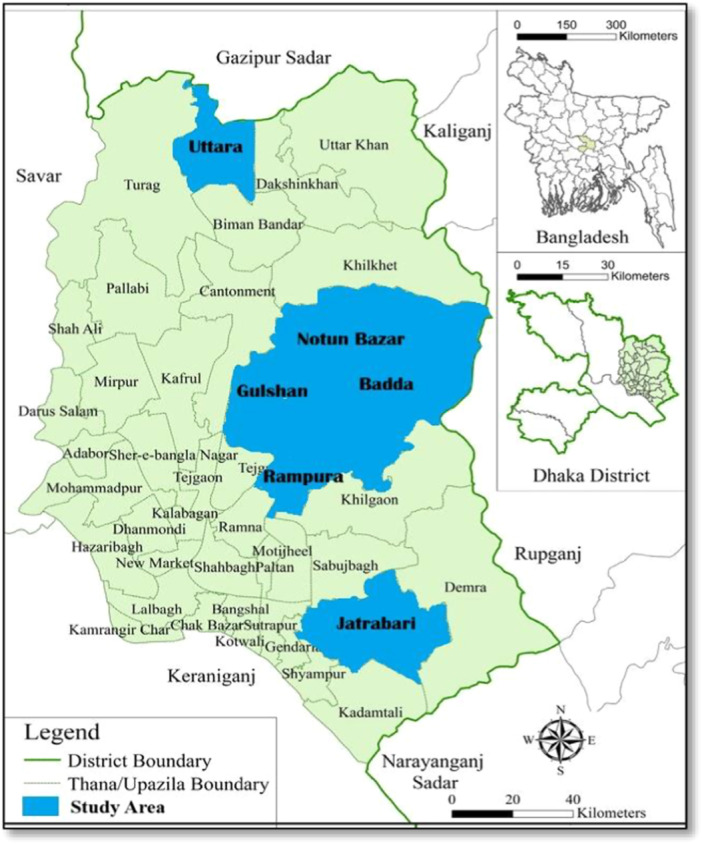
Map showing the selected study areas under Dhaka North City Corporation (DNCC) and Dhaka South City Corporation (DSCC) where data were collected from urban waste collectors.

The research was carried out in some selected locations under DNCC and DSCC, which are the two administrative authorities responsible for municipal solid waste management in Dhaka City. Six areas, including Uttara, Gulshan, Badda, Notun Bazar, Rampura, and Jatrabari, were purposively selected because of their high population density, diverse waste streams, and heavy reliance on both formal and informal waste collectors (Figure 1). Inclusion of areas from within both City Corporations made it possible to capture variation in working environments and waste management practices.

### 2.3 Study Population and Sampling

Participants were identified and approached directly at waste collection points, roadside cleaning locations, and waste transfer areas where workers were actively engaged in waste management activities. Due to the informal and mobile nature of waste collection work in Dhaka City, it was not possible to establish a complete sampling frame. Therefore, participants were recruited using purposive sampling based on their availability and willingness to participate at the time of data collection. The study population comprised urban waste collectors engaged in city corporation, contractual, and informal waste collection activities, including household waste collection, street cleaning, and drain or manhole cleaning. Both male and female workers were included in order to reflect the gender composition of the sector. According to City Corporation estimates, several thousand workers are involved in waste collection activities across Dhaka North and South City Corporations. A total of 40 waste collectors were included in the study. Inclusion criteria included workers aged 18 years or older who were actively engaged in waste collection at the time of the survey.

### 2.4 Data Collection

Data were collected through face‐to‐face semi‐structured interviews conducted at respondents' work locations. The interview guide was developed based on prior literature on occupational health and waste management. The questionnaire items were informed by previous studies examining occupational health risks among waste collectors and sanitation workers in LMICs [6, 10, 13].

The interview guide covered the following domains:

- Sociodemographic characteristics
- Occupational characteristics and working hours
- Types of waste handled and work activities
- Use of PPE
- Occupational safety training and waste‐sorting practices
- Self‐reported work‐related health problems
- Awareness of occupational health risks
- Access to healthcare services and institutional support

Each interview lasted approximately 30–45 min and was conducted in Bangla, the preferred language of the participants. Before data collection, the study objectives were explained and verbal informed consent was obtained from all respondents. Privacy and confidentiality were maintained throughout the data collection process.

### 2.5 Measurement of Health Insecurity

Health insecurity was measured by using a Health Insecurity Index, which was created to reflect the accumulation of self‐reported work‐related health problems among waste collectors.

#### 2.5.1 Variables Included

The index included the following health issues that are often linked to working in rubbish collection:

- Respiratory disease
- Skin infections
- Allergic conditions
- Itching‐related problems
- Work injuries
- Diabetes

#### 2.5.2 Scoring Method and Range

Each health condition was coded as a binary variable (1 = present, 0 = absent). The Health Insecurity Index score for each respondent was calculated as the sum of the individual condition scores. The possible score ranged from 0 to 6, with higher scores indicating greater cumulative health insecurity.

#### 2.5.3 Interpretation and Validation

The index was analyzed as a continuous outcome in correlation and regression analyses. Because this was a small exploratory study, the index was not externally validated and internal consistency testing (e.g., Cronbach's *α*) was not performed. The index should therefore be interpreted as a descriptive composite indicator of cumulative vulnerability rather than as a validated diagnostic scale. Similar composite indicators have been used in exploratory occupational health research where validated instruments are not available.

### 2.6 Data Analysis

Data were reviewed, coded, entered into Microsoft Access for quality checks, and then exported for statistical analysis in IBM SPSS Statistics version 26.0 (IBM Corp., Armonk, NY, USA). Microsoft Excel was used for preliminary data cleaning and for preparing tables. Statistical reporting was revised in line with the SAMPL recommendations and the guidance of Assel et al. [18] where applicable. Categorical variables were summarized as counts and percentages (*n*/*N*), and key proportions are presented with 95% confidence intervals when informative.

Inferential analyses were conducted as follows:

- *χ* ^2^ testing was used to assess the association between PPE use and disease occurrence. To avoid reporting *p* values alone, the comparison is also described using observed risks and effect estimates.

- Pearson's correlation coefficients were used to examine the strength and direction of the relationships between working hours, PPE use, occupational training, waste‐sorting practices, and the Health Insecurity Index.
- Exploratory multiple linear regression was used to examine the association of working hours, PPE use, occupational training, and waste‐sorting practices with the Health Insecurity Index. Unstandardized coefficients (*B*), standard errors, standardized coefficients (*β*), and *p* values are reported.
- Given the relatively small sample size and the number of predictors included, the regression analysis was treated as exploratory. The authors acknowledge the possibility of limited statistical power and model overfitting and interpretation of the regression results should therefore be made with caution as indicative associations and not considered definitive predictors. All statistical tests were two‐tailed and a significance level of *p* < 0.05 was considered statistically significant.

The reporting of study design, sampling procedures, statistical analysis, and study limitations followed the Strengthening the Reporting of Observational Studies in Epidemiology recommendations for cross‐sectional studies where applicable.

### 2.7 Ethical Considerations

The study was conducted in 2025 in accordance with the ethical principles for human‐participant research. Ethical approval was obtained from the Ethical Clearance Committee (ECC) of Dhaka International University (Approval No: DIU/RPC/ECC/04/SOC/007, Date: 10/04/2025). Verbal informed consent was obtained from all respondents before interview, participation was voluntary, respondents could withdraw at any time without consequence, and confidentiality was maintained by removing personal identifiers from the data set.

## 3 Results

This section presents the key findings of the study based on the survey conducted among 40 urban waste collectors in Dhaka City. Descriptive statistics for the main study variables, including sociodemographic characteristics, occupational factors, working hours, and safety practices, are presented in Tables 1, 2, 3. Subsequent sections present inferential statistical analyses examining associations between occupational factors and the Health Insecurity Index.

**Table 1:** Sociodemographic and occupational characteristics of urban waste collectors (*N* = 40).

Variable	Category	*n*	%
Gender	Male	31	77.5
	Female	9	22.5
Age (years)	< 30	21	52.5
	30–40	12	30
	40–50	5	12.5
	> 50	2	5
Monthly income (BDT)	5000–8000 (≈USD 42–67)	5	12.5
	9000–12,000 (≈USD 75–100)	10	25
	13,000–15,000 (≈USD 108–125)	20	50
	> 15000 (> USD 125)	5	12.5
Working hours/day	5–7 h	6	15
	8–10 h	27	67.5
	> 11 h	7	17.5
Type of waste handled	Household waste collection	18	45
	Street cleaning	9	22.5
	Industrial waste collection	5	12.5
	Drainage cleaning and manhole maintenance	8	20
Employment category	Informal collectors	18	45
	Formal collectors	22	55

**Table 2:** Descriptive statistics and subgroup comparisons among urban waste collectors (*N* = 40).

Variable	Overall (*N* = 40)	Male (*n* = 31)	Female (*n* = 9)	Informal (*n* = 18)	Formal (*n* = 22)
Age (years)	31.4 ± 9.2	31.6 ± 9.0	30.8 ± 9.8	30.9 ± 9.4	32.7 ± 8.8
Monthly income	BDT 11,500 ± 2800 (≈USD 96 ± 23)	BDT 11,700 ± 2700 (≈USD 98 ± 23)	BDT 10,900 ± 2900 (≈USD 91 ± 24)	BDT 11,200 ± 2900 (≈USD 93 ± 24)	BDT 12,100 ± 2400 (≈USD 101 ± 20)
Working hours/day	9.2 ± 1.8	9.3 ± 1.7	8.8 ± 1.9	9.5 ± 1.7	8.9 ± 1.6

*Note:* Values are presented as mean ± SD. USD values calculated using 1 USD ≈ 120 BDT. Subgroup comparisons are presented descriptively due to the relatively small sample size.

**Table 3:** Use of personal protective equipment, occupational safety training, and waste‐sorting practices among respondents (*N* = 40).

Variable	Category	*n*	%
Use of personal protective equipment (PPE)	Yes	12	30
	No	28	70
Occupational safety training	Yes	9	22.5
	No	31	77.5
Waste‐sorting practices	Yes	6	15
	No	34	85

*Note:* Field Survey, 2025.

### 3.1 Sociodemographic and Occupational Characteristics

The sociodemographic and occupational characteristics of the respondents are summarized in Table 1. A total of 40 waste collectors participated in the study, of whom 31 (77.5%) were male and 9 (22.5%) were female, indicating that waste collection activities are predominantly performed by men. The age distribution shows that waste collection is largely carried out by a relatively young workforce. More than half of the respondents (21/40, 52.5%) were younger than 30 years, followed by 12 respondents (30.0%) aged between 30 and 40 years. Only a small proportion of respondents were older than 40 years (7/40, 17.5%).

Monthly income levels were generally low, reflecting the economic vulnerability of waste collectors. Half of the respondents (20/40, 50.0%) reported earning between BDT 13,000 and 15,000 per month (approximately USD 108–125), while 10 respondents (25.0%) reported incomes between BDT 9000 and 12,000 (approximately USD 75–100). The estimated mean monthly income was BDT 11,500 ± 2800 (approximately USD 96 ± 23).

Working hours among the respondents were relatively long. Most workers (27/40, 67.5%) reported working 8–10 h/day, while 7 respondents (17.5%) reported working more than 11 h daily. The average working duration was 9.2 ± 1.8 h/day, indicating substantial occupational exposure to waste collection activities. Although the majority of waste collectors were male (31/40, 77.5%), female workers (9/40, 22.5%) were also represented in the sample. Descriptive comparisons suggested broadly similar occupational exposure patterns between male and female respondents; however, the relatively small number of female participants limited the possibility of detailed subgroup statistical analysis.

The majority of the workers were involved in household waste collection (18/40, 45.0%), followed by street cleaning (9/40, 22.5%), industrial waste collection (5/40, 12.5%), and drainage cleaning and manhole maintenance (8/40, 20.0%). Based on the employment category, informal collectors made up 45.0% of the respondents (18/40), while formal collectors accounted for 55.0% (22/40).

These findings indicate that informal waste collectors were more frequently engaged in household and street‐level waste collection activities, while formal collectors were typically associated with city corporation waste management services. This structure reflects the dual nature of the waste management system in Dhaka, where both formal city corporation waste management services and a large informal waste collection sector exist side by side.

Overall, these findings highlight the socioeconomic vulnerability of waste collectors and the occupational environments that may contribute to their health risks.

Table 2 summarizes the descriptive statistics of the main variables included in the study. The average working duration among respondents was 9.2 ± 1.8 h/day, while the mean monthly income was BDT 11,500 ± 2800 (≈USD 96 ± 23).

### 3.2 Waste‐Sorting Practices, Safety Measures, and Training

Waste‐sorting practices among respondents indicate limited adherence to occupational safety measures (Table 3). Only 12/40 workers (30.0%) reported using PPE, while 70.0% (28/40) reported working without any protective gear. Similarly, only 9/40 respondents (22.5%) reported receiving formal occupational safety training. Waste‐sorting practices were also uncommon, reported by 6/40 workers (15.0%).

These findings suggest that most waste collectors operate under conditions of frequent exposure to mixed waste streams without adequate protective measures or safety training, reflecting significant gaps in occupational safety practices and institutional support.

### 3.3 Association Between PPE Use and Disease Occurrence

The association between PPE use and reported disease occurrence was examined using a *χ* ^2^ test (Table 4). Disease was reported by 26 of 28 workers (92.9%) who did not use PPE, compared with 6 of 12 workers (50.0%) who reported PPE use (Table 4). Pearson's *χ* ^2^ test indicated a statistically significant association between PPE use and disease occurrence (*χ* ^2^ = 9.87, df = 1, *p* = 0.002). Fisher's exact test also confirmed the significance of this association (*p* = 0.005).

**Table 4:** Association between PPE use and disease occurrence (*N* = 40).

PPE use	Disease present *n* (%)	Disease absent *n* (%)	Total
Yes	6 (50.0)	6 (50.0)	12
No	26 (92.9)	2 (7.1)	28
Total	32 (80.0)	8 (20.0)	40

*Note:* Chi‐square test: *χ* ^2^ = 9.87, df = 1, *p* = 0.002. Fisher's exact test: *p* = 0.005. Risk ratio = 1.86 (95% CI: 1.04–3.30).

Based on the risks calculated from Table 4, the estimated risk ratio was 1.86 (95% CI: 1.04–3.30), suggesting that workers who did not use PPE had a higher likelihood of reporting disease compared with those who used protective equipment. However, given the cross‐sectional design of the study, these findings should be interpreted as associations rather than causal relationships.

### 3.4 Health Problems and Health Insecurity

Work‐related health problems were widely reported among the respondents (Table 5). Respiratory disease was reported by 35/40 workers (87.5%), followed by itching‐related dermatological conditions (87.5%), skin infections (75.0%), allergic conditions (62.5%), and work‐related injuries (25.0%). A smaller proportion reported chronic conditions such as diabetes (12.5%).

**Table 5:** Self‐reported health problems among waste collectors (*N* = 40).

Health problem	*n*	%
Respiratory disease	35	87.5
Skin infections	30	75
Allergic conditions	25	62.5
Itching‐related dermatological problems	35	87.5
Work‐related injuries	10	25
Diabetes	5	12.5

*Note:* Field Survey, 2025.

These conditions were combined into a Health Insecurity Index (range: 0–6) representing the cumulative burden of self‐reported health problems. Higher index scores indicate greater health insecurity and occupational vulnerability among waste collectors.

#### 3.4.1 Awareness of Health Risks Associated With Waste Collection

The level of health‐risk awareness among respondents is presented descriptively. Specifically, 75% (30/40 individuals) reported having health awareness, suggesting a relatively high level of knowledge regarding disease prevention and related health practices. However, 25% (10/40 individuals) stated that they do not have such awareness, which is a large minority who may fall at a higher risk of having health complications due to lack of information or access to health education.

### 3.5 Correlation Between Occupational Factors and Health Insecurity

Pearson's correlation analysis was conducted to examine the relationships between occupational factors and the Health Insecurity Index (Table 6). Working hours showed a significant positive correlation with the Health Insecurity Index (*r* = 0.58, *p* < 0.01).

**Table 6:** Pearson's correlation matrix between occupational factors and Health Insecurity Index (*N* = 40).

Variables	1	2	3	4	5
1. Health Insecurity Index	1				
2. Working hours	0.58**	1			
3. PPE use	−0.62**	−0.4**	1		
4. Occupational training	−0.41**	−0.35*	0.48**	1	
5. Waste‐sorting practices	−0.33*	−0.29	0.39*	0.44**	1

*Note:* Pearson's correlation coefficients are presented.

* *p* < 0.05;

** *p* < 0.01.

In contrast, PPE use (*r* = −0.62, *p* < 0.01), occupational training (*r* = −0.41, *p* < 0.01), and waste‐sorting practices (*r *= −0.33, *p* < 0.05) were negatively correlated with the Health Insecurity Index. These findings indicate that longer working hours were associated with higher levels of health insecurity, whereas engagement in protective practices and safety training was associated with lower levels of reported health risk.

### 3.6 Exploratory Regression Analysis of Health Insecurity

An exploratory multiple linear regression analysis was conducted to examine potential predictors of the Health Insecurity Index (Table 7). The regression model explained 56% of the variance in health insecurity (*R* ^2^ = 0.56, adjusted *R* ^2^ = 0.52, *F*(4,35) = 11.94, *p* < 0.001).

**Table 7:** Regression analysis predicting Health Insecurity Index (*N* = 40).

Independent variables	*B*	Std. error	*β*	*t*	*p*
Constant	1.87	0.42	—	4.45	< 0.001
Working hours	0.43	0.09	0.45	4.78	< 0.001
PPE use (Yes = 1)	−0.39	0.11	−0.38	−3.55	0.001
Training (Yes = 1)	−0.31	0.14	−0.29	−2.21	0.03
Waste sorting (Yes = 1)	−0.24	0.13	−0.21	−1.84	0.07

*Note:* Model summary: *R* ^2^ = 0.56, adjusted *R* ^2^ = 0.52, *F*(4,35) = 11.94, *p* < 0.001. *B* indicates the unstandardized regression coefficient; *β* indicates the standardized regression coefficient.

Working hours were positively associated with the Health Insecurity Index (*β* = 0.45, *p* < 0.001). In contrast, PPE use (*β* = −0.38, *p* = 0.001) and occupational safety training (*β* = −0.29, *p* = 0.03) were significantly associated with lower levels of health insecurity. Waste‐sorting practices also showed a negative association but did not reach the conventional significance threshold (*p* = 0.07).

Given the relatively small sample size, these findings should be interpreted as exploratory associations rather than definitive predictors.

Overall, the statistical analyses suggest that occupational factors such as working hours, PPE use, and occupational safety training may be important correlates of health insecurity among urban waste collectors.

### 3.7 Access to Healthcare Services and Institutional Support

Access to institutional healthcare services among waste collectors was limited (Table 8). Only 5/40 respondents (12.5%) reported having access to health facilities through institutional arrangements, while the majority (87.5%) reported no such access. None of the respondents reported receiving financial support for health‐related needs.

**Table 8:** Access to institutional health facilities and financial support among respondents (*N* = 40).

Variable	Category	*n*	%
Provision of health facilities	Yes	5	12.5
	No	35	87.5
Financial support	Yes	0	0
	No	40	100

*Note:* Field Survey, 2025.

These findings indicate a substantial lack of institutional healthcare provision and social protection for urban waste collectors, potentially increasing their vulnerability to occupational health risks.

Overall, the results indicate that urban waste collectors in Dhaka experience a substantial burden of work‐related health problems alongside limited access to occupational safety measures and institutional support. Several occupational factors, including working hours, PPE use, and occupational safety training, showed statistically significant associations with the Health Insecurity Index. However, given the exploratory nature of the analysis and the relatively small sample size, these findings should be interpreted with caution. The following discussion section interprets these results in relation to existing literature and broader occupational health contexts.

## 4 Discussion

To our knowledge, this study provides one of the few quantitative assessments of occupational health insecurity among urban waste collectors in Dhaka City and contributes to the limited empirical evidence on occupational health risks among waste workers in Bangladesh. The findings indicate that waste collectors experience a substantial burden of work‐related health problems and operate in occupational environments characterized by limited safety measures and institutional support. The study further identifies key occupational and institutional factors associated with increased health vulnerability in this workforce, providing preliminary evidence that may inform future research and policy interventions aimed at improving occupational health conditions among waste collectors. These findings highlight the importance of strengthening occupational health systems for waste collectors, who play a critical but often overlooked role in maintaining urban sanitation and public health.

### 4.1 Occupational Health Burden Among Urban Waste Collectors

The results indicate that urban waste collectors in Dhaka experience a considerable burden of work‐related health problems. As presented in Table 5, respiratory diseases, dermatological conditions, allergic reactions, and work‐related injuries were widely reported among the respondents. Respiratory disease and itching‐related dermatological conditions were reported by 87.5% of workers, while skin infections and allergic conditions were also common. Similar patterns of occupational health risks have been documented among waste workers in other LMICs, where exposure to dust, contaminated materials, and bioaerosols has been associated with respiratory illnesses, skin infections, and musculoskeletal disorders [6, 11, 12, 13].

Waste collectors frequently handle unsegregated household, industrial, and occasionally medical waste, which may contain hazardous chemicals, infectious materials, and sharp objects. Previous studies have identified these exposures as key contributors to occupational health risks among sanitation workers in LMIC settings [11, 12]. Therefore, the high prevalence of health problems observed in this study likely reflects broader occupational conditions within informal and semi‐formal waste management systems.

### 4.2 PPE Use and Disease Risk

The results also demonstrate a significant association between the use of PPE and disease occurrence among waste collectors. As shown in Table 4, workers who did not use PPE reported substantially higher disease prevalence compared with those who used protective equipment. The *χ* ^2^ analysis indicated a statistically significant association between PPE use and disease occurrence, and the estimated risk ratio suggested a higher likelihood of disease among workers who did not use PPE.

These findings are consistent with previous research showing that inadequate use of PPE increases exposure to biological and chemical hazards during waste handling activities [11, 13]. Studies conducted among sanitation workers in several developing countries have also shown that the consistent use of gloves, masks, and protective clothing can significantly reduce the risk of respiratory infections, skin diseases, and occupational injuries [12, 14]. The relatively low prevalence of PPE use observed in this study, therefore, highlights an important gap in occupational safety practices within the waste management sector in Dhaka.

### 4.3 Working Hours and Occupational Exposure

The statistical analyses further indicate that occupational exposure duration may be an important factor influencing health insecurity. The correlation analysis (Table 6) showed that working hours were positively associated with the Health Insecurity Index, indicating that longer working hours were linked to higher levels of cumulative health risk. This association was also supported by the regression analysis (Table 7), where working hours remained a significant predictor of health insecurity.

Extended working hours may increase cumulative exposure to hazardous waste materials, dust, and bioaerosols. Similar findings have been reported in previous studies where prolonged occupational exposure among sanitation workers was associated with higher risks of respiratory illnesses, musculoskeletal disorders, and occupational injuries [6, 12]. Waste collection activities often involve repetitive physical movements, manual lifting of heavy loads, and prolonged contact with contaminated materials, which may cumulatively increase occupational health risks. However, given the relatively small sample size and exploratory nature of the regression model, these associations should be interpreted cautiously and viewed as indicative rather than definitive causal relationships.

### 4.4 Role of Occupational Safety Training and Protective Practices

The correlation and regression analyses also indicate that protective occupational practices may reduce health insecurity among waste collectors. As shown in Tables 6 and 7, PPE use and occupational safety training were negatively associated with the Health Insecurity Index. Workers who reported using protective equipment or receiving occupational safety training tended to report lower levels of health insecurity.

Previous research has similarly highlighted the importance of occupational safety training in improving workers' awareness of workplace hazards and promoting safer waste‐handling practices [13, 14]. Training programs may enhance workers' knowledge regarding the proper use of protective equipment, safe waste handling procedures, and hygienic practices. However, the findings of this study show that only a small proportion of workers reported receiving formal safety training (Table 3), suggesting that occupational safety education remains limited within the waste management sector.

### 4.5 Institutional and Structural Challenges

The study findings also highlight broader institutional challenges related to occupational health protection for waste collectors. As shown in Table 3, most workers reported limited access to PPE and occupational safety training. In addition, Table 8 indicates that access to institutional healthcare services was very limited among the respondents, and none of the workers reported receiving financial support for health‐related needs.

Similar institutional gaps have been documented in other LMIC contexts, where waste collectors frequently work within informal or semi‐formal labour arrangements that provide limited access to occupational health protection and social security benefits [11, 14]. Weak enforcement of OHS regulations may further contribute to unsafe working conditions in the waste management sector.

### 4.6 Public Health and Environmental Implications

Improving occupational health conditions for waste collectors has broader implications for public health and environmental sustainability. Waste collectors play a critical role in maintaining urban sanitation systems and preventing environmental contamination. Previous studies have demonstrated that effective waste management contributes significantly to disease prevention, environmental protection, and urban public health outcomes [15, 17].

Unsafe working conditions not only threaten the health of waste collectors but may also undermine the efficiency and sustainability of urban waste management systems. Strengthening occupational safety measures, improving waste segregation practices, and enhancing institutional support for waste workers could therefore contribute to both worker protection and improved urban environmental health.

### 4.7 Study Limitations

Several limitations of this study should be acknowledged. First, the sample size was relatively small and limited to selected areas of Dhaka City, which may restrict the generalizability of the findings. The relatively small sample size reflects the exploratory nature of the study and the practical challenges associated with identifying and recruiting waste collectors working in informal urban environments. Second, the cross‐sectional design does not allow causal relationships to be established between occupational factors and health outcomes. Third, health conditions were self‐reported and may therefore be subject to recall or reporting bias.

Because participants were recruited through purposive sampling in informal work settings, the study may be subject to selection bias. Workers who were more accessible or willing to participate may differ from those who were not included in the survey. Therefore, the findings may not be fully representative of all waste collectors in Dhaka City and should be interpreted as exploratory evidence. Future research involving larger sample sizes and longitudinal study designs would provide more robust evidence regarding occupational health risks among waste collectors. Despite these limitations, the study provides valuable exploratory insights into occupational health vulnerabilities among urban waste collectors in Dhaka, a population for which systematic empirical data remain scarce.

### 4.8 Policy Implications

Despite these limitations, the findings of this study provide important insights for strengthening occupational health policies in the waste management sector. City Corporation authorities and waste management institutions should prioritize the provision of adequate PPE, implement regular occupational safety training programs, and ensure access to healthcare services for waste collectors. Strengthening waste segregation practices and improving enforcement of OHS regulations may also reduce occupational health risks among waste workers and contribute to safer and more sustainable urban waste management systems [13, 15].

## 5 Conclusion

This study provides empirical evidence on occupational health insecurity among urban waste collectors in Dhaka City and highlights several occupational and institutional factors associated with increased health vulnerability in this workforce. The findings indicate a substantial burden of work‐related health problems among waste collectors, particularly respiratory illnesses, dermatological conditions, and allergic reactions. Limited use of PPE, lack of occupational safety training, and prolonged working hours were associated with higher levels of health insecurity.

Although the findings should be interpreted cautiously due to the relatively small sample size and cross‐sectional design, the study contributes to the limited quantitative evidence on occupational health risks among waste collectors in Bangladesh. Strengthening occupational safety measures, improving access to PPE and training, and expanding institutional health support may help reduce occupational health risks and improve the well‐being of waste collectors in rapidly urbanizing cities. Future research involving larger samples and longitudinal study designs would help to further clarify the relationships between occupational exposures and health outcomes in this vulnerable population.

## 6 Recommendations

Based on the findings of this study, several measures can be recommended to improve occupational health conditions among urban waste collectors:

1. City Corporation authorities should ensure the regular supply and mandatory use of appropriate PPE.
2. Regular occupational safety training programs should be implemented to increase awareness of workplace hazards and safe waste‐handling practices.
3. Waste segregation at the source should be strengthened to reduce exposure to hazardous materials.
4. Periodic health monitoring and access to healthcare services should be provided for waste collectors.
5. Institutional mechanisms and regulatory enforcement of OHS standards should be strengthened.

## Author Contributions

**Kamrunnahar Koli:** conceptualization, data collection, analysis, and manuscript writing. **Salma Begum:** supervision, methodology development, and manuscript review. **MD. Abujar Gifary Efat:** data interpretation, statistical support, and manuscript editing. All authors have read and approved the final version of the manuscript.

## Funding

The authors have nothing to report.

## Disclosure

The lead author Kamrunnahar Koli affirms that this manuscript is an honest, accurate, and transparent account of the study being reported; that no important aspects of the study have been omitted; and that any discrepancies from the study as planned (and, if relevant, registered) have been explained.

## Ethics Statement

Ethical approval was obtained from the Ethical Clearance Committee (ECC) of Dhaka International University (Approval No: DIU/RPC/ECC/04/SOC/007, Date: 10/04/2025).

## Consent

Verbal informed consent was obtained from all respondents before interview.

## Conflicts of Interest

The authors declare no conflicts of interest.

## Data Availability

The data supporting the findings of this study are available from the corresponding author upon reasonable request. The data set contains anonymized survey responses and does not include personally identifiable information. Kamrunnahar Koli had full access to all of the data in this study and takes complete responsibility for the integrity of the data and the accuracy of the data analysis.
